# Risk factors and outcomes in non-transplant patients with extended-spectrum beta-lactamase-producing *Escherichia coli* bacteremia: a retrospective study from 2013 to 2016

**DOI:** 10.1186/s13756-019-0599-y

**Published:** 2019-08-27

**Authors:** Tingting Xiao, Kai Yang, Yanzi Zhou, Shuntian Zhang, Jinru Ji, Chaoqun Ying, Ping Shen, Yonghong Xiao

**Affiliations:** State Key Laboratory for Diagnosis and Treatment of Infectious Disease, Collaborative Innovation Center for Diagnosis and Treatment of Infectious Diseases, the First Affiliated Hospital, College of Medicine, Zhejiang University, Hangzhou, China

**Keywords:** Non-transplantation, Extended-spectrum β-lactamase, *Escherichia coli*, Bloodstream infection, Prognosis

## Abstract

**Background:**

*Escherichia coli* is one of the most common strains of extended-spectrum β-lactam (ESBL)-producing bacteria, and the prevention and treatment of ESBL-producing *E. coli* infections is an ongoing challenge. The clinical characteristics and outcomes of ESBL-producing *E. coli* bacteremia in non-transplant patients remain to be elucidated.

**Methods:**

This retrospective study included 491 non-transplant patients with *E. coli* bloodstream infections (BSIs) from January 2013 to December 2016 and was conducted to investigate the risk factors, clinical features, and outcomes of these infections.

**Results:**

Of the 491 *E. coli* BSI patients, 57.6% suffered from infections with ESBL-producing strains. A multivariate analysis showed that urinary tract infection, prior use of cephalosporin, and treatment with β-lactam-β-lactamase inhibitor (BLBLI) combination antibiotics were independent risk factors for the development of ESBL-producing *E. coli* BSIs. The overall mortality rate in *E. coli* BSI patients was 14.46%, and there was no significant difference in the 28 day mortality rate between ESBL-producing *E. coli* and non-ESBL-producing *E. coli* BSI patients (14.8% vs. 14.0%, respectively; *P* = 0.953). Similarly, there was no difference between the community-acquired infection group and the nosocomial infection group. Hepatobiliary disease, carbapenem exposure, high APACHE II score, and hypoproteinemia were independent risk factors for death in *E. coli* BSI patients. Multivariate analysis showed that hypoproteinemia and severe disease were independent risk factors for death from *ESBL*-producing *E. coli* BSIs. Furthermore, there was no significant difference in the 28 day mortality between patients with *ESBL*-producing *E. coli* BSIs treated with carbapenem monotherapy versus those treated with BLBLI combination antibiotics (12.8% vs. 17.9%, respectively; *P* = 0.384).

**Conclusions:**

Prior use of cephalosporin or BLBLI combination antibiotics increased the risk ratio for *ESBL*-producing *E. coli* infection. Hypoproteinemia and severe disease are independent risk factors for death in patients with *E. coli* BSIs. There was no significant difference in the 28 day prognosis of patients with ESBL-producing *E. coli* and those with non-ESBL-producing *E. coli* BSIs. These data do not support the conclusion that carbapenems might be more effective than BLBLI antibiotics for treatment of patients with BSIs caused by ESBL-producing *E. coli*.

**Electronic supplementary material:**

The online version of this article (10.1186/s13756-019-0599-y) contains supplementary material, which is available to authorized users.

## Background

*Escherichia coli* is one of the most common strains of extended-spectrum β-lactamase (ESBL)-producing bacteria [1]. Since the first *ESBL*-producing bacterial strains were discovered in the 1980s, new genotypes have frequently emerged [2]. In the past 10 years, the detection rate of ESBL-producing *E. coli* has continued to increase dramatically worldwide. In Asia, and particularly in China, the prevalence rate is much higher than that in Western developed countries [3, 4]. The production of β-lactamase enzymes is the principal mechanism by which Gram-negative bacteria resist the action of β-lactam antibiotics. According to relevant domestic monitoring data, *E. coli* accounted for the largest proportion of resistant bacteria detected in China, followed by *K. pneumoniae* [5].

Previous studies have shown that prior use of broad-spectrum antibiotics, poor general condition, advanced age, and some invasive procedures (such as central venous catheterization) are risk factors for ESBL-producing *E. coli* infection [6, 7]. For patients with ESBL-producing *E. coli* bloodstream infections (BSIs), improved prognoses depend on a detailed understanding of the factors that increase mortality. Increased mortality due to inappropriate antibiotic treatment (whether empirical or definite) has been widely reported in previous studies. Furthermore, other risk factors (e.g., poor general health condition, liver disease, septic shock, and antibiotic exposure) prior to infection have also been reported in the literature [8, 9].

ESBL-producing *E. coli* represents a serious public health issue and infection control challenge, and the selection of appropriate treatments for ESBL-producing *E. coli* BSIs remains controversial as there are insufficient large-scale randomized controlled trials to support various therapeutic approaches. Carbapenem antibiotics are still considered to be first-line antibiotics for treating ESBL-producing *E. coli* BSIs [10]. However, in recent years, widespread use of these antibiotics has driven the development of carbapenem-resistant *Enterobacteriaceae* (CRE). Available monitoring data showed that the resistance rate of Enterobacteriaceae to β-lactam antibiotics such as cefotaxime, ceftazidime, cefepime, and piperacillin-tazobactam decreased from 2005 to 2014, while the meropenem (2005, 2.8%; 2014, 4.5%) and ertapenem (2005, 5.5%; 2014, 8.9%) resistance rates showed an increasing trend [11]. Currently, a growing number of experts believe that there is a wealth of data showing that the effectiveness of treatment with β-lactam-β-lactamase inhibitor (BLBLI) combination antibiotics is similar to that of carbapenems in patients infected with ESBL-producing strains. Antibiotic drugs such as cephalosporins and BLBLI combination antibiotics (e.g., piperacillin-tazobactam) have once again gradually attracted attention.

It is worth noting that ESBL-producing *E. coli* bacteremia occurs often in solid organ transplant recipients, which may be due to their frequent hospitalization, longer hospital stays, and long-term exposure to immunosuppressive agents. Moreover, some studies have shown that these patients have higher ESBL-producing *E. coli* infection and mortality rates [12–14]. To the best of our knowledge, no studies have analyzed the risk and prognosis of non-transplanted ESBL-producing *E. coli* BSIs; therefore, in this study, patients with solid organ and hematopoietic stem cell transplants were excluded.

This retrospective analysis was designed to investigate the clinical characteristics of non-transplant patients with ESBL-producing *E. coli* infections as well as to analyze the risk and prognostic factors and the therapeutic effects of different antibiotic regimens with the goals of strengthening our understanding of ESBL-producing *E. coli* infections and providing new guidance for clinical practice.

## Methods

### Population

This study retrospectively analyzed the clinical and microbiological data of patients with BSIs caused by *Escherichia coli* in the First Affiliated Hospital of Zhejiang University from January 2013 to December 2016 after receiving approval from the research ethics committee. Data from patients with the following characteristics were included in the analysis: a) a blood culture positive for *E. coli*, b) clinical manifestations of infection, and c) hospitalization for more than 48 h with a complete clinical data set. Patients aged < 16 years and those without complete medical records were excluded. Patients with histories of organ or hematopoietic stem cell transplantation before the BSI and those with carbapenem-resistant *E. coli* infections were excluded. If the same patient had more than one BSI within 6 months, only data from the first BSI were included. Ultimately, based on the inclusion and exclusion criteria, 491 patients were included in the study.

### Bacterial identification and drug sensitivity testing

The VITEK 2 COMPACT automatic microbial identification system was used for bacterial identification and drug susceptibility testing. According to the guidelines of the Clinical and Laboratory Standards Institute (CLSI) standards (2015) [15], ESBL production was determined using a double-disk potentiation test with amoxicillin-clavulanic acid and cefotaxime, ceftazidime, or cefepime or ESBL-positive results from the VITEK-2 N131 analysis. Carbapenem resistance was defined as a minimum inhibitory concentration (MIC) of ≥2 mg/L for ertapenem or a MIC of ≥4 mg/L for imipenem or meropenem.

### Data collection

Demographic and clinical data were collected from the electronic case system, including age, gender, underlying disease, comorbidities, length of hospital stay, admission to the ICU, invasive procedures before and after infection, and antibiotic treatments. The severity of the disease was assessed via the Acute Physiology and Chronic Health Evaluation (APACHE) II scores and Pitt scores [16]. The Charlson index was used to assess the burden of any comorbidities [17]. A three-part analysis was conducted. First, risk factors associated with ESBL-producing *E. coli* infection were evaluated by comparing the ESBL-producing and non-ESBL-producing patient groups. Second, to investigate the risk factor of mortality, the 491 *E. coli* BSI patients were divided into survival and death groups according to their survival status after 28 days of infection. Finally, 283 patients with ESBL-producing *E. coli* BSIs were analyzed to assess the risk factors associated with 28 day mortality and various antibiotic treatments.

### Definitions

*E. coli* BSI was defined as an infection manifested by the presence in at least one blood culture that grew a *E. coli* strain. BSI refers to the systemic inflammatory response syndrome caused by toxins and metabolites produced by pathogens. Possible sources of *E. coli* BSIs are based on the Centers for Disease Control (CDC) and Prevention/National Healthcare Safety Network (NHSN) surveillance definitions [18]. Glucocorticoid therapy was defined as prednisolone > 20 mg/day for more than 7 days. Antimicrobial drug exposure referred to the use of antibiotics for > 72 h 30 days prior to the BSI diagnosis. Empirical therapy was defined as a therapeutic drug administered at the time of the blood culture test or before the blood culture report based on clinical experience. Definitive therapy referred to antimicrobial therapy administered based on drug susceptibility results. Treatments were classified as “appropriate” if the regimen contained at least one drug effective against *E. coli*; otherwise, they were classified as “inappropriate”. All-cause mortality was defined as death from an *E. coli* BSI within 7, 14 and 28 days of the onset of bacteremia.

### Data analysis

In the univariate analysis of ESBL-producing *E. coli* BSIs, the χ^2^ or Fisher’s two-tailed test was used for categorical variables, and Student’s t test (for variables with normal distributions) and the Mann-Whitney U test (for variables with non-normal distributions) were used for continuous variables. For continuous variables, the results are expressed as mean ± standard deviation (SD) or median (interquartile range [IQR]). For categorical variables, the percentages for each group are reported. Variables with *P* values ≤0.05 in the univariate analysis were included in the multivariate analysis, and binary logistic regression (backward: condition) was used to identify independent predictors. The survival distribution functions in different groups were compared via the Kaplan-Meier product limit method. All data were statistically analyzed using SPSS version 23.0, and *P* values ≤0.05 were considered statistically significant.

## Results

### Clinical characteristics

From January 2013 to December 2016, 491 patients with *E. coli* BSIs were included in this study, except for 67 patients who received solid organ or hematopoietic stem cell transplants and 22 patients with CRE BSIs. Among the included patients, 283 (57.6%) had ESBL-producing *E. coli* BSIs and 208 (42.4%) had non-ESBL-producing *E. coli* BSIs. There were no significant differences in age and gender between the two groups (60.8 ± 16.7 vs. 60.5 ± 16.3, *P* = 0.836; male, 112 vs. 150, *P* = 0.853). Of the primary infection sites of the 491 patients with *E. coli* BSIs, abdominal infections were the highest (*n* = 249), followed by respiratory infections (*n* = 108) and urinary tract infections (*n* = 84). The annual incidence rates of ESBL-producing *E. coli* BSIs from 2013 to 2016 were 66.2% (86/130), 59.1% (88/149), 49.1% (53/108), and 53.8% (48/104), respectively, and while the incidence rate fluctuated, it did not increase significantly (*P* = 0.638).

### Risk factors for ESBL-producing *E. coli* BSIs

A univariate analysis (Table 1) showed that risk factors for ESBL-producing *E. coli* BSIs included urinary system infection, prior surgery, prior invasive procedures (central venous catheterization, gastric catheterization, and percutaneous catheterization), and antibiotic use within 30 days before infection (mainly cephalosporins and BLBLI combination regimens). In a multivariate analysis, logistic regression analysis showed that urinary tract infections were the primary site of *E. coli* BSIs (OR = 1.897, *P* = 0.014) and that cephalosporin exposure (OR = 2.767, *P* = 0.007) and treatment with BLBLI combination regimens in the 30 days prior to the BSI (OR = 1.950, *P* = 0.010) were independent risk factors for ESBL-producing *E. coli* BSIs.

**Table 1:** Clinical and Demographic Characteristics of Patients with BSI Caused by *E. coli*

	Univariate analysis			Multivariable analysis			
	*Non- ESBL- producing E. coli*	*ESBL- producing E. coli*	*P*-values	*P*-values	OR	95% CI for OR	
	(*n* = 208)	(*n* = 283)				Lower	Upper
Demographic							
Male, n (%)	112 (53.8)	150 (53.0)	0.853				
Age, mean ± SD	60.8 ± 16.7	60.5 ± 16.3	0.836				
Total hospital stay, days (median, IQR)	19 (11–28)	18 (11–37)	0.188				
Hospital stay before BSI, days (median, IQR)	3 (0–13)	4 (1–14)	0.336				
ICU stay prior to BSI^a^	11 (5.3)	25 (8.8)	0.136				
Preexisting medical conditions							
Hypertension	58 (27.9)	80 (28.3)	0.925				
Diabetes	34 (16.4)	45 (15.9)	0.876				
Lung disease	2 (1.0)	6 (2.1)	0.477				
Cardiovascular diseases	4 (1.9)	7 (2.5)	0.921				
Hepatobiliary disease	59 (28.4)	78 (27.6)	0.844				
Urinary system disease	15 (7.2)	25 (8.8)	0.516				
Nervous system disease	7 (3.4)	8 (2.8)	0.732				
Malignant solid tumor	43 (20.7)	63 (22.3)	0.673				
Hematological Disease	39 (18.8)	35 (12.4)	0.051				
Charlson comorbidity score ^c^ (median, IQR)	2 (0–2)	2 (0–2)	0.168				
Likely source of BSI							
Central vein Catheter-related infections	7.2 (7.2)	10 (3.5)	0.067				
Lung infection	52 (25.0)	56 (19.8)	0.168				
Abdominal infection	108 (51.9)	141 (49.8)	0.646				
Urinary infection	26 (12.5)	58 (20.5)	0.020	0.014	1.897	1.138	3.164
Intracranial infection	1 (0.5)	4 (1.4)	0.402				
Skin infection	6 (2.9)	11(3.9)	0.548				
Primary bloodstream infection	19 (9.1)	26 (9.2)	0.984				
Nosocomial-acquired infection	124 (59.6)	180 (63.6)	0.368				
Hospitalization prior to BSI^b^	75 (56.0)	110 (59.1)	0.571				
Surgery prior to BSI^a^	27 (13.0)	56 (19.8)	0.047				
Invasive procedure and/or devices prior to BSI^a^	46 (22.1)	83 (29.3)	0.073				
Mechanical ventilation	7 (3.4)	11 (3.9)	0.761				
Central venous catheterization	20 (9.6)	42 (14.8)	0.085				
Urinary catheterization	18 (8.7)	42 (14.8)	0.039				
Gastric catheterization	10 (4.8)	31(11.0)	0.015				
Percutaneous catheterization	6 (2.9)	21 (7.4)	0.029	0.101	2.218	0.857	5.742
Hemodialysis prior to BSI^a^	6 (2.9)	3 (1.1)	0.251				
Chemotherapy or radiotherapy prior to BSI^a^	38 (18.3)	39 (13.8)	0.177				
Corticosteroid use prior to BSI^a^	15 (7.2)	21 (7.4)	0.930				
Immunosuppressant use prior to BSI^a^	1 (0.5)	2 (0.7)	> 0.050				
Antibiotics use prior to BSI^a^	68 (32.7)	134 (47.3)	0.001				
Cepholosporins	10 (4.8)	38 (13.4)	0.001	0.007	2.767	1.325	5.779
BLBLI combination antibiotics	26 (12.5)	62 (21.9)	0.007	0.010	1.950	1.173	3.241
Tigecycline	2 (1.0)	3 (1.1)	> 0.050				
Carbapenems	17 (8.2)	20 (7.1)	0.646				
Aminoglycosides	1 (0.5)	2 (0.7)	> 0.050				
Quinolones	26 (12.5)	36 (12.7)	0.942				
Laboratory examination							
White blood cell	8.1 (3.8–13.0)	9.6 (5.5–14.8)	0.045				
Platelet (median, IQR)	104 (42–183)	117 (58–192)	0.161				
Serum total protein (median, IQR)	57.7 (52.0–64.7)	58.3 (52.3–64.3)	0.949				
Severity of illness							
APACHEII score (median, IQR)	9 (6–12)	9 (6–13)	0.540				
Appropriate empirical treatment after BSI	200 (96.2)	249 (88.3)	0.001				
Appropriate definitive treatments after BSI	204 (98.1)	267 (94.3)	0.039				
7-day mortality	17 (8.2)	24 (8.1)	0.985				
14-day mortality	23 (11.1)	28 (9.9)	0.676				
28-day mortality	29 (13.9)	42 (14.8)	0.780				

Data are expressed as n (%) unless otherwise stated

*Abbreviations*: *ESBL* extended-spectrum beta-lactamase; *β-lactam-β-lactamase inhibitor* (BLBLI) combination antibiotics, *APACHE* acute physiology and chronic health evaluation, *BSI* bloodstream infection, *ICU* intensive care unit, *IQR* interquartile range, *SD* standard deviation

^a^During the 30 days preceding BSI onset

^b^During the 3 months preceding BSI onset

^c^At time of BSI onset

### Risk factors affecting the mortality of patients with *E. coli* infection

**Fig. 1:**
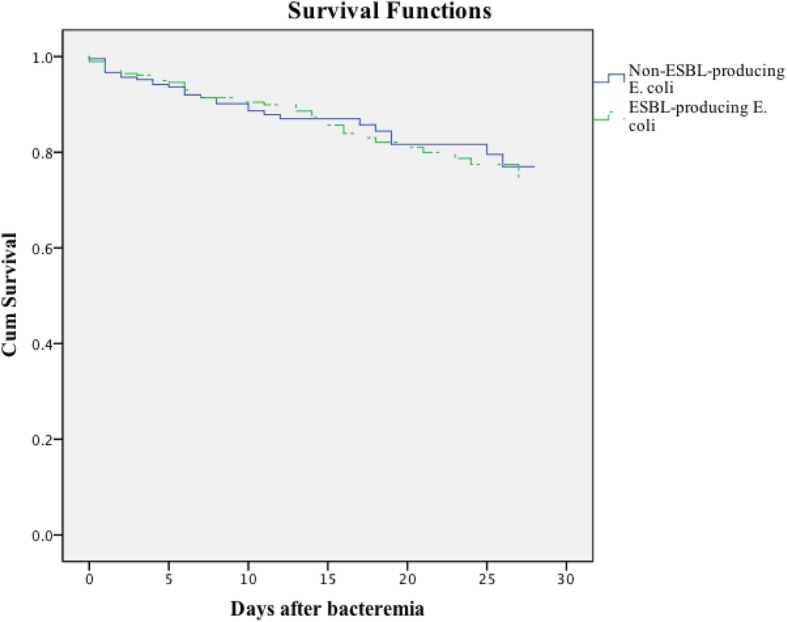
Kaplan-Meier survival estimates among patients with BSIs caused by non-ESBL-producing *E. coli* and ESBL-producing *E. coli*

The non-transplanted patients with *E. coli* BSIs were classified into survivor and death groups based on the outcome at 28 days. The overall all-cause 28 day mortality rate of the 491 patients was 14.46% (71/491). A survival curve analysis (Fig. 1) showed that the mortality of the patients in the ESBL-producing *E. coli* group was higher than that of the patients in the non-ESBL-producing *E. coli* group (14.9% vs. 14.2%, χ^2^ = 0.003, *P* = 0.953), and the difference between the mortalities was not statistically significant. Furthermore, the patients were divided into community infection and nosocomial infection groups (Additional file 1: Figure S1). In 187 community-infected BSI patients (8.4%), there was no statistically significant difference in the mortality between those with non-ESBL-producing *E. coli* infections and those with ESBL-producing *E. coli* infections (8.3% vs. 11.7%; χ^2^ = 0.472, *P* = 0.492). There was also no significant difference in the mortalities between these two groups in the patients with nosocomial infections (17.7% vs. 16.7%; χ^2^ = 0.240, *P* = 0.624).

The main characteristics of the *E. coli* BSI survivor and non-survivor subgroups are shown in Table 2. Based on a multivariate regression analysis, the following factors were independently associated with a higher mortality risk in patients with *E. coli* BSIs: hepatobiliary disease (OR = 1.890, *P* = 0.034), prior use of carbapenem antibiotics (OR = 2.839, *P* = 0.016), and higher APACHE II scores (OR = 1.313, *P* < 0.001). Normal serum total protein (OR = 0.959, *P* = 0.006) and appropriate definite antibiotic treatment were independent protective factors (OR = 2.777, *P* = 0.017) for the outcomes of patients with *E. coli* BSIs.

**Table 2:** Analysis of risk factors for 28-day Mortality in 491 patients with *E. coli* bloodstream infections

	Univariate analysis			Multivariable analysis			
	Survivors (*n* = 420)	Non- survivors (*n* = 71)	*P*-values	*P*-values	OR	95% CI for OR	
						Lower	Upper
Demographic							
Male, n (%)	223 (53.1)	39 (54.9)	0.774				
Ages, mean ± SD	60.8 ± 16.4	60.0 ± 16.8	0.736				
Total hospital stay, days (median, IQR)	20 (11–34)	15 (7–28)	0.013				
Hospital stay before BSI, days (median, IQR)	3 (0–13)	7 (1–17)	0.028				
Preexisting medical conditions							
Hypertension	115 (27.4)	23 (32.4)	0.385				
Diabetes	71 (16.9)	8 (11.4)	0.249				
Lung disease	5 (1.2)	3 (4.2)	0.173				
Cardiovascular diseases	10 (2.4)	1 (1.4)	0.937				
Hepatobiliary disease	110 (26.2)	27 (38.0)	0.040	0.034	1.890	1.048	3.410
Urinary system disease	34 (8.1)	6 (8.5)	0.919				
Nervous system disease	13 (3.1)	2 (2.8)	> 0.050				
Malignant solid tumor	86 (20.5)	20 (28.2)	0.145				
Hematological Disease	63 (15.0)	11 (15.5)	0.914				
Charlson comorbidity score ^b^ (median, IQR)	2 (0–2)	2 (1–3)	0.001				
Source of infections							
Central venous catheterization	25 (6.0)	0 (0)	0.069				
Lung infection	82 (19.5)	26 (36.6)	0.001	0.071	1.781	0.951	3.336
Abdominal infection	212 (50.5)	37 (52.1)	0.799				
Urinary infection	74 (17.6)	10 (14.1)	0.464				
Intracranial infection	4 (1.0)	1 (1.4)	0.544				
Skin infection	15 (3.6)	2 (2.8)	> 0.050				
Primary bloodstream infection	39 (9.3)	6 (8.5)	0.822				
Nosocomial- acquired infection	252 (60.0)	52 (73.2)	0.034				
ESBL-producing E. coli, n (%)	241 (57.4)	42 (59.2)	0.780				
ICU stay prior to BSI ^a^	30 (7.1)	6 (8.5)	0.696				
ICU stay after BSI ^c^	32 (7.6)	11 (15.5)	0.030				
Prior surgery ^a^	69 (16.4)	14 (19.7)	0.494				
Surgery after BSI ^c^	37 (8.8)	2 (2.8)	0.084				
Invasive procedure and/or devices prior to BSI ^a^	106 (25.2)	23 (32.4)	0.205				
Mechanical ventilation	14 (3.3)	4 (5.6)	0.540				
Central venous catheterization	48 (11.4)	14 (19.7)	0.052				
Urinary catheterization	47 (11.2)	13 (18.3)	0.090				
gastric catheterization	33 (7.9)	8 (11.3)	0.337				
Percutaneous catheterization	24 (5.7)	3 (4.2)	0.820				
Invasive procedure and/or devices after BSI ^c^	67 (16.0)	13 (18.3)	0.619				
Mechanical ventilation	29 (6.9)	14 (19.7)	< 0.001				
Central venous catheterization	108 (25.7)	32 (45.1)	0.001				
Urinary catheterization	103 (24.5)	27 (38.0)	0.017				
Gastric catheterization	73 (17.4)	19 (26.8)	0.061				
Percutaneous catheterization	47 (11.2)	7 (9.9)	0.740				
Hemodialysis prior to BSI ^a^	8 (1.9)	1 (1.4)	> 0.050				
Chemotherapy or radiotherapy prior to BSI ^a^	65 (15.5)	12 (16.9)	0.760				
Corticosteroid use prior to BSI ^a^	28 (6.7)	8 (11.3)	0.169				
Hemodialysis after BSI ^c^	14 (3.3)	3 (4.2)	0.977				
Corticosteroid use after BSI ^c^	32 (7.6)	6 (8.5)	0.808				
Prior Antibiotics use ^a^	164 (39)	38 (53.5)	0.022				
Cepholosporins	43 (10.2)	5 (7.0)	0.402				
BLBLI combination antibiotics	72 (17.1)	16 (22.5)	0.273				
Tigecycline	3 (0.7)	2 (2.8)	0.321				
Carbapenems	25 (6.0)	12 (16.9)	0.001	0.016	2.839	1.215	6.635
Aminoglycosides	2 (0.5)	1 (1.4)	0.375				
Quinolones	52 (12.4)	10 (14.0)	0.689				
Laboratory examination ^b^							
White blood cell (median, IQR)	9.1 (5.1–14.2)	9.6 (3.7–13.3)	0.849				
Platelet (median, IQR)	117 (52–192)	80 (37–177)	0.078				
Total protein (median, IQR)	58.9 (53.0–64.7)	53.9 (46.0–62.0)	< 0.001	0.006	0.959	0.931	0.988
Severity of illness at time of BSI ^b^							
APACHEII score (median, IQR)	9 (6–12)	13 (9–17)	< 0.001	< 0.001	1.131	1.071	1.195
Appropriate empirical treatment after BSI ^c^	389 (92.6)	60 (84.5)	0.024				
1. Cepholosporins	25 (6.0)	3 (4.2)	0.761				
2. BLBLI combination antibiotics	150 (35.7)	25 (35.2)	0.935				
3. Carbapenems	189 (45.0)	34 (47.9)	0.651				
4. Quinolones	49 (11.7)	6 (8.5)	0.427				
5. Aminoglycosides	12 (2.9)	2 (2.8)	> 0.050				
6. Tigecycline	5 (1.2)	2 (2.8)	0.598				
Appropriate definitive treatments after BSI ^c^	407 (96.9)	64 (90.1)	0.008	0.017	2.777	1.198	6.437

Data are expressed as n (%) unless otherwise stated

*Abbreviations*: *ESBL* extended-spectrum beta-lactamase; *β-lactam-β-lactamase inhibitor* (BLBLI) combination antibiotics, *APACHE* acute physiology and chronic health evaluation, *BSI* bloodstream infection, *ICU* intensive care unit, *IQR* interquartile range, *SD* standard deviation

^a^During the 30 days preceding BSI onset

^b^At time of BSI onset

^c^After BSI onset

### Risk factors for mortality in ESBL-producing *E. coli* BSIs and treatment regimens

A total of 283 ESBL-producing *E. coli* BSI patients were included in this analysis, and the 28 day mortality was 14.8%. In the univariate analysis (Additional file 2: Table S1), pulmonary infection, invasive procedures and/or device implementation before and after development of a BSI, antibiotic use within 30 days prior to infection, hypoproteinemia, and higher APACHE II scores were risk factors for increased mortality in patients with ESBL-producing *E. coli* BSIs. A multivariate analysis revealed that hypoproteinemia (OR = 0.941, *P* = 0.045) and higher APACHE II scores (OR = 1.103, *P* = 0.003) were independent risk factors for poor outcomes.

As shown in Additional file 2: Table S1, of the patients infected with ESBL-producing *E. coli*, 87.99% (249/283) received appropriate empirical antibiotic therapy and 94.35% (267/283) received appropriate definite antibiotic therapy. Appropriate empirical antibiotic treatment was a protective factor in the univariate analysis, and the rate of patients receiving appropriate empirical antibiotic treatment in the death group was significantly lower than that in the survival group (78.2% vs. 89.6%, *P* = 0.042). Based on our multivariate analysis, appropriate empirical antibiotic treatment also appeared to be a protective factor that can reduce the mortality rate, although this effect was marginally significant (OR = 2.526, *P* = 0.058).

**Fig. 2:**
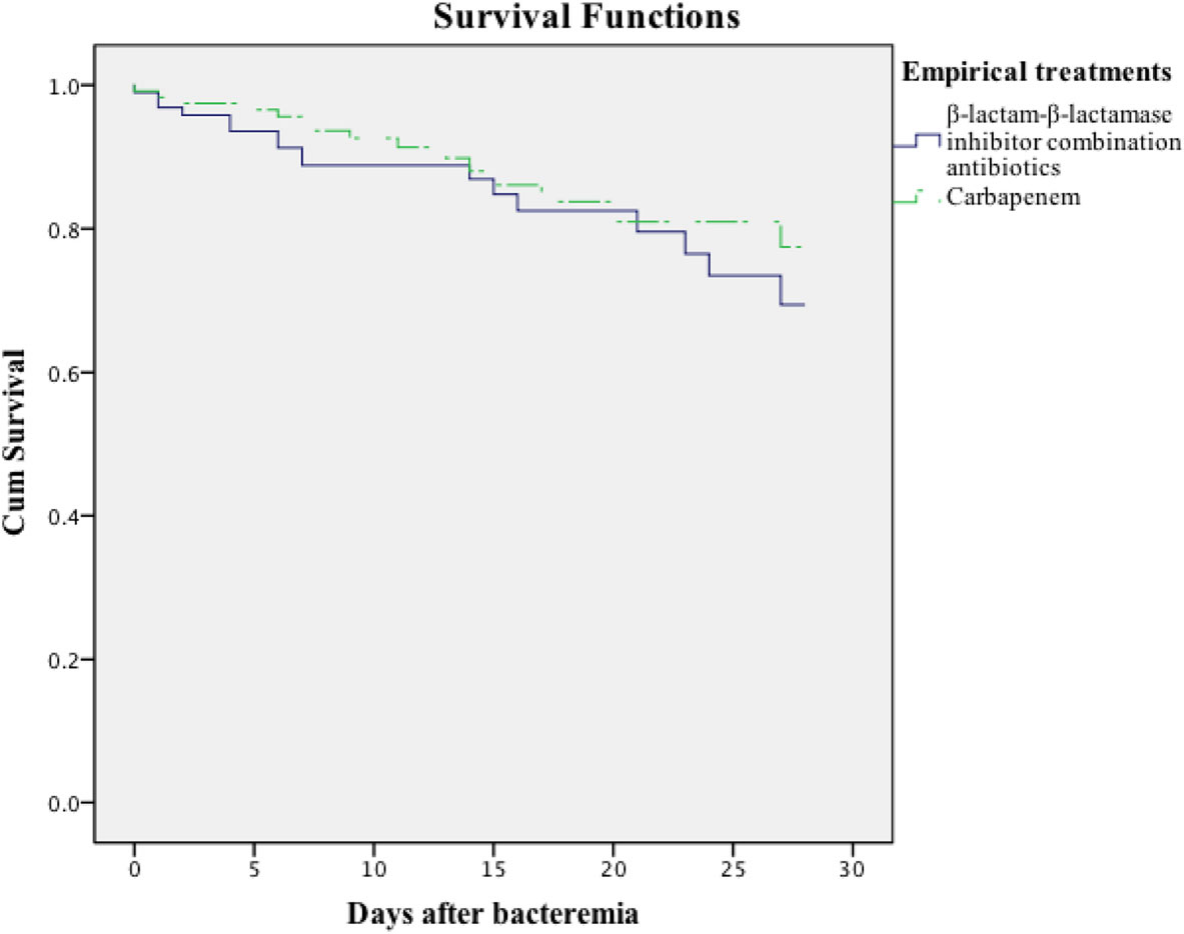
Kaplan-Meier 28 day survival estimates among ESBL-producing *E. coli* BSI patients depending on treatment with carbapenem or β-lactam-β-lactamase inhibitor (BLBLI) combination antibiotics

In terms of empirical treatment, 117 patients with ESBL-producing *E. coli* BSIs were treated with carbapenem monotherapy, and 95 patients were treated with BLBLI combination monotherapy regimens. Figure 2 shows that a single-antibiotic regimen with carbapenem (*n* = 117) or a BLBLI regimen (*n* = 95) resulted in no differences in 28 day mortality between the two groups (12.8% vs. 17.9%; χ^2^ = 0.759, *P* = 0.384), and their APACHE II scores were 10 and 9, respectively. Considering the potential confounding effect of severity, the patients were divided into two groups according to their APACHE II scores (< 9 and ≥ 9), and the difference in the prognoses of the two groups was compared (Additional file 1: Figure S2). For patients with APACHE II scores ≥9 at the onset of bacteremia, there was no statistically significant difference in the 28 day mortality rate between the patients who received carbapenem and those who received BLBLI monotherapy (16.4% vs. 22.4%; χ^2^ = 0.249, *P* = 0.617); similarly, no significant difference was observed between the two groups in patients with APACHE II scores < 9 (6.8% vs. 10.8%; χ^2^ = 0.694, *P* = 0.405).

## Discussion

*Escherichia coli* is one of the most common pathogens in clinical infections. The primary mechanism of drug resistance in these bacteria is the production of ESBLs, and this mechanism underlies a high proportion of the antibiotic resistance cases in China [5]. This study focused on studying the risk factors associated with ESBL-producing *E. coli* BSIs in non-transplanted patients.

Antimicrobial use prior to BSI is believed to be an important factor in drug-resistant infections [19, 20], although some studies have shown no association between ESBL-producing *E. coli* infections and prior antibiotic therapy; for example, Denis et al. reported that there was no significant difference in the prevalence of antibiotic exposure between the ESBL-producing *E. coli* and non-ESBL-producing *E. coli* groups, which included 82 patients (*P* = 0.32) [21]. Our results also demonstrated that cephalosporins and BLBLI combination regimens within 30 days prior to infection were independent risk factors for ESBL-producing *E. coli* BSIs, which is consistent with the findings of many previous studies. Regarding the likely infectious source of *E. coli* BSIs, intra-abdominal infections were most common, followed by respiratory tract infections and urinary tract infections. However, most studies showed that ESBL-producing *E. coli* strains are primarily derived from urinary tract infections [7, 9, 22], followed by respiratory and bloodstream infections. Furthermore, we found that patients with nosocomial infections accounted for 50% of the patients with *E. coli* urinary tract infections (*n* = 29) and 66% of the patients with intra-abdominal infections (*n* = 93); therefore, this significant difference between the frequencies of patients with infections in these two sites is likely due to a higher likelihood of acquiring an abdominal infection in the hospital. Based on this observation, we suspect that the reason for the high proportion of intra-abdominal infections in this study may be related to the high number of patients undergoing hepatology-related procedures and hepatobiliary surgery in our hospital. It is well known that *E. coli* is an opportunistic bacterial pathogen that can invade the body when invasive procedures disrupt the mucosa. The multivariate analysis of the risk factors associated with ESBL-producing *E. coli* in this study showed that urinary tract infection was an independent risk factor (*P* = 0.014), consistent with previous studies [23–25]. Currently, ESBL-producing *E. coli* BSIs originating from urinary tract infections are more commonly studied, while articles focusing on abdominal infections are rare, suggesting a further research direction.

To explore the high mortality rate associated with *E. coli* BSIs, we evaluated the patient characteristics and the treatments they received. In this study, there was no significant difference in the 28 day mortality between patients infected with ESBL-producing *E. coli* and those infected with non-ESBL-producing *E. coli* (*P* = 0.953), similar to the findings of some previous studies [21, 26]. Conversely, other studies demonstrated significantly higher mortality in patients infected with ESBL-producing *E. coli* than in those infected with non-ESBL-producing *E. coli* [6]. A possible reason for the similar mortality rates in the two groups in the present study is the use of many broad-spectrum antibiotics due to the current high prevalence of ESBL-producing *E. coli*. For patients with end-stage liver disease, bacterial infection is one of the most common causes of death [27], which explains the observation that hepatobiliary system disease was an independent risk factor for mortality in the present study. Furthermore, an analysis of patients with potential liver disease and *E. coli* infection by Kang et al. yielded similar results [28]. Our analysis showed that disease severity is also an independent risk factor for mortality in patients with ESBL-producing *E. coli* BSIs, which is also similar to the results of previous studies [29]. We also found that low serum albumin is an independent risk factor for death in patients with *E. coli* and ESBL-producing *E. coli* BSIs. Serum albumin level is a common indicator for assessing a patient’s nutritional status, organ function, and comorbidity. The inflammatory state resulting from bacterial infection, which leads to the production of IL-1, TNF, and other cell mediators, can interfere with liver albumin synthesis, resulting in hypoalbuminemia [30]. There is currently a lack of literature on the relationship between mortality and serum albumin level in patients with *E. coli* infections. Akirov et al. [31] studied the relationship between serum albumin levels and prognosis in hospitalized patients (without any detailed division) and found that low albumin levels were positively correlated with short- and long-term mortality in the entire hospitalized patient population. Albumin levels are often lower in patients with hepatic insufficiency, which was also associated with higher mortality in the patients with hepatobiliary disease included in this study. Du et al. assessed the outcomes of 85 patients with *E. coli* infections and found that prior use of BLBLI combination antibiotics increased the mortality rate [32]. In the present study, we also found that use of carbapenems within 30 days prior to infection was an important independent risk factor for the patients who died, which has been rarely reported previously. Previous studies [33] have shown that short-term use of such antibiotics before infection may also lead to the production of and infection by carbapenem-resistant bacteria. These outcomes increase the complexity of treating critical infections, which might explain the increased mortality observed in this study.

In terms of the antibiotic options for treating *E. coli* BSIs, we believe that appropriate, defined antibiotic treatments are a protective factor for reducing death due to *E. coli* BSIs, as reported in this study (OR = 2.777, *P* = 0.017), and this observation is consistent with those of some other studies [7, 34]. The findings of attempts to determine the best treatment options for ESBL-producing *E. coli* have been inconclusive, although carbapenems have long been considered the best antibiotic for treating infections with ESBL-producing *E. coli* [35]. However, the wide use of carbapenems has been associated with the emergence of CRE. In recent years, the efficacy advantages of carbapenems over other antibiotics have been increasingly questioned. The latter antibiotics include BLBLI combination antibiotics, cefepime, and quinolones (among others), and the feasibility of using the above-mentioned older antibiotics to treat ESBL-producing *E. coli* infections is becoming more and more accepted by researchers [36–38]. We analyzed the outcomes of 283 patients with ESBL-producing *E. coli* BSIs, including 95 patients with BLBLI combination monotherapy regimens and 117 with carbapenem monotherapy (median APACHE II 9 and 10). Their 28 day mortality rates were 12.8 and 17.9%, respectively, and the difference was not statistically significant (χ^2^ = 0.759, *P* = 0.384). Because disease severity may affect prognoses, we divided the ESBL-producing *E. coli* BSI patients into two groups according to their APACHE II scores (< 9, ≥ 9) and assessed their 28 day mortality rates. We found no statistically significant difference in 28 day mortality between the two groups (Additional file 1: Figure S2 *P* = 0.405; *P* = 0.617), which confirms that carbapenem antibiotics are no longer more effective than BLBLI combination antibiotics against ESBL-producing *E. coli* BSIs. These findings are consistent with previous reports that proposed the use of BL/BLIs to treat ESBL-producing *E. coli* BSIs [22, 39]. For example, Muhammed et al. [39] conducted a meta-analysis of more than 1000 patients in 14 relevant articles and found that there was no significant difference in prognosis between patients treated with carbapenems and those treated with BLBLI combination antibiotics for either empirical or definite antibiotic treatment. However, some reports support the opposite opinion that carbapenem antibiotics can significantly reduce mortality compared with other antibiotics [40]. Currently, as stated above, it remains unclear in all of the relevant studies whether carbapenems were primarily administered to patients with more comorbidities or to those with more severe clinical conditions.

Regarding the limitations of this study, our findings are based mainly on retrospective data and studies, which makes it impossible to eliminate certain types of bias. Furthermore, while this study excluded patients with solid organ and hematopoietic stem cell transplantation, some patients with underlying diseases may have been treated with immunosuppressive agents. Finally, we were unable to perform further stratification of the outcomes based on patient genotyping due to a lack of phenotypic testing. In this context, we should perform more detailed drug resistance gene detection and drug susceptibility testing in ESBL-producing *E. coli* BSI patients to better understand the drug resistance profiles involved as well as the relevant treatment options.

## Conclusion

In summary, prior exposure to cephalosporin and BLBLI combination antibiotics increases the risk of acquiring ESBL-producing *E. coli* infections. Prior carbapenem use and a poor systemic condition were risk factors for increased mortality in patients with ESBL-producing *E. coli* and non-ESBL-producing *E. coli* BSIs; however, no significant difference was found in the prognoses of patients with ESBL-producing *E. coli* and non-ESBL-producing *E. coli* BSIs. Hypoproteinemia and severe disease lead to worse outcomes for patients with ESBL-producing *E. coli* BSIs. Carbapenem offered no significant advantage over BLBLI combination antibiotics for improving the 28 day mortality of patients with ESBL-producing *E. coli* BSIs.

## Additional files

Additional file 1: **Figure S1.** Kaplan-Meier survival estimates among non-transplant patients with BSIs caused by non-ESBL-producing *E. coli* and ESBL-producing *E. coli*. (A) Community-acquired infection. (B) Nosocomial-acquired infection. **Figure S2.** Kaplan-Meier 28 day survival estimates. (A) *E. coli* BSI patients (APACHE II score < 9) treated with carbapenem and β-lactam-β-lactamase inhibitor (BLBLI) combination antibiotics. (B) *E. coli* BSI patients (APACHE II score ≥ 9) treated with carbapenem and BLBLIs. (DOCX 122 kb)

Additional file 2: **Table S1.** Analysis of risk factors for 28 day mortality in 283 patients with ESBL- producing *E. coli* BSI. (DOCX 31 kb)

## Data Availability

Full datasets analysed during the current study are available from the corresponding author on reasonable request.

## Abbreviations

APACHE: Acute Physiology and Chronic Health Evaluation
BLBLI: β-lactam-β-lactamase inhibitor combination antibiotics
BSI: Bloodstream infection
*E. coli*: *Escherichia coli*
ESBL: Extended-spectrum beta-lactamase
ICU: Intensive care unit
IQR: Interquartile range
SD: Standard deviation
