# Supplementary material for: Risk factors and outcomes in non-transplant patients with extended-spectrum beta-lactamase-producing Escherichia coli bacteremia: a retrospective study from 2013 to 2016
Source: Antimicrob Resist Infect Control. 2019 Aug 27;8:144. doi: 10.1186/s13756-019-0599-y (PMC6712786; doi:10.1186/s13756-019-0599-y)

**Figure S1. Kaplan-Meier survival estimates among non-transplant patients with BSIs caused by non-ESBL-producing *E. coli* and ESBL-producing *E. coli*. (A) Community-acquired infection (8.3% vs. 11.7%; χ^2^=0.472, P=0.492). (B) Nosocomial-acquired infection (17.7% vs. 16.7%; χ^2^=0.240, P=0.624).**

**Figure S2. Kaplan-Meier 28 day survival estimates. (A) *E. coli* BSI patients (APACHE II score < 9) treated with carbapenem and β-lactam-β-lactamase inhibitor (BLBLI) combination antibiotics (3/44 vs. 4/37; χ^2^=0.694, P=0.405). (B) *E. coli* BSI patients (APACHE II score** **≥ 9) treated with carbapenem and BLBLIs (12/73 vs. 13/58; χ^2^=0.249, P=0.617).**

**Figure S1. Kaplan-Meier survival estimates among non-transplant patients with BSIs caused by non-ESBL-producing *E. coli* and** **ESBL-producing *E. coli*.**

**(A) Community-acquired infection**

**(B)** **Nosocomial****-acquired infection**


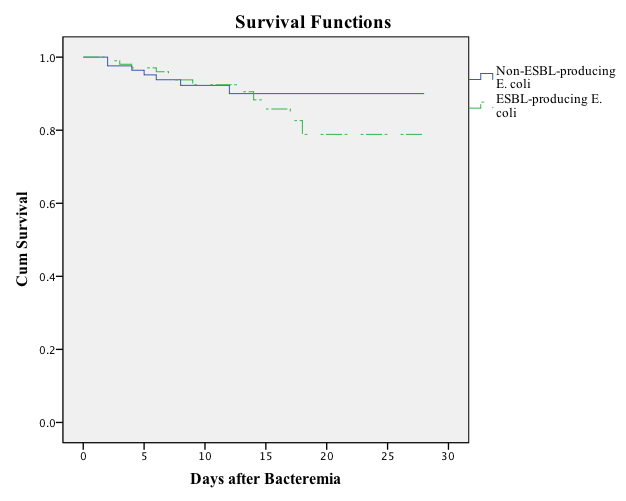

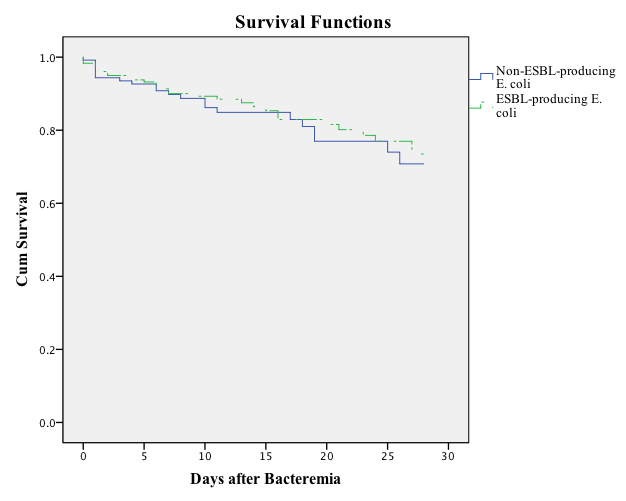


**Figure S2. Kaplan–Meier 28-day survival estimates:**

**(A)** ***E. coli*-BSI patients (APACHE II score< 9) treated with** **carbapenem and** **β-lactam-β-lactamase inhibitor (BLBLI) combination antibiotics;**

**(B) *E. coli*-BSI patients (APACHE II score≥9) treated with** **carbapenem and BLBLIs**


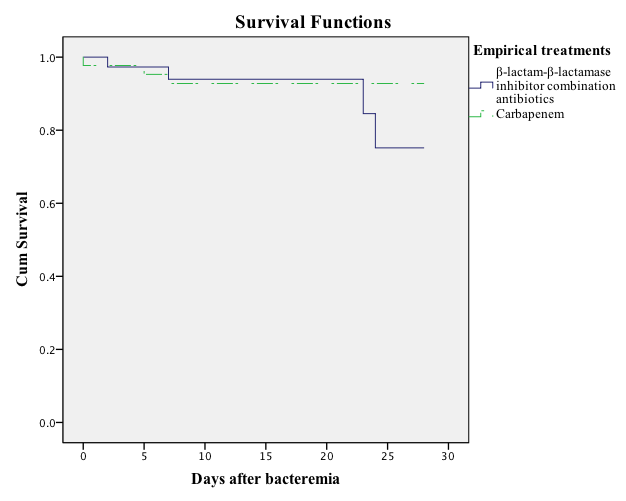

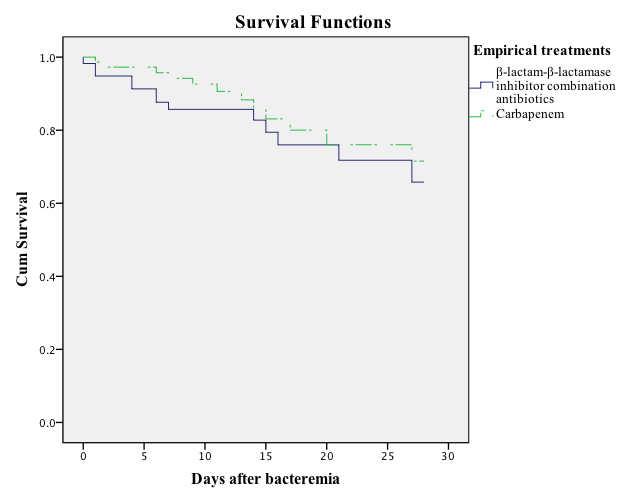

Supplement: Supplementary file 1 — Figure S1. Kaplan-Meier survival estimates among non-transplant patients with BSIs caused by non-ESBL-producing E. coli and ESBL-producing E. coli. (A) Community-acquired infection. (B) Nosocomial-acquired infection. Figure S2. Kaplan-Meier 28 day survival estimates. (A) E. coli BSI patients (APACHE II score < 9) treated with carbapenem and β-lactam-β-lactamase inhibitor (BLBLI) combination antibiotics. (B) E. coli BSI patients (APACHE II score ≥ 9) treated with carbapenem and BLBLIs. (DOCX 122 kb) [file 13756_2019_599_MOESM1_ESM.docx]
