# Supplementary material for: Risk factors and outcomes in non-transplant patients with extended-spectrum beta-lactamase-producing Escherichia coli bacteremia: a retrospective study from 2013 to 2016
Source: Antimicrob Resist Infect Control. 2019 Aug 27;8:144. doi: 10.1186/s13756-019-0599-y (PMC6712786; doi:10.1186/s13756-019-0599-y)
Supplement: Supplementary file 2 — Table S1. Analysis of risk factors for 28 day mortality in 283 patients with ESBL- producing E. coli BSI. (DOCX 31 kb) [file 13756_2019_599_MOESM2_ESM.docx]

**Table S1 Analysis of risk factors for 28 day mortality in 283 patients with ESBL- producing *E. coli* BSI**

|  | | | |  | Univariate analysis | | | Multivariable analysis | | | |
| --- | --- | --- | --- | --- | --- | --- | --- | --- | --- | --- | --- |
|  | | | |  | Survivors  (*n* =241) | Non- survivors  (*n* = 42) | *P*-  values | *P*-  values | OR | 95% CI for OR | |
|  |  |  |  |  |  |  |  |  |  |  | |
|  |  |  |  |  |  |  |  |  |  | Lower | Upper |
| Demographic | | | | |  |  |  |  |  |  |  |
|  | | Male, n (%) | | | 129 (53.5) | 21 (50.0) | 0.673 |  |  |  |  |
|  | | Ages, mean ± SD | | | 60.3±16.1 | 61.9±17.6 | 0.551 |  |  |  |  |
| Total hospital stay, days (median, IQR) | | | | | 19 (12-37) | 4 (0-14) | 0.182 |  |  |  |  |
| Hospital stay before BSI, days (median, IQR) | | | | | 17 (9-35) | 8 (1-19) | 0.063 |  |  |  |  |
| ICU stay prior to BSI ^a^ | | | | | 22 (9.1) | 3 (7.1) | 0.901 |  |  |  |  |
| Preexisting medical conditions | | | | |  |  |  |  |  |  |  |
|  | Hypertension | | | | 63 (26.1) | 17 (40.5) | 0.057 |  |  |  |  |
|  | Diabetes | | | | 40 (16.6) | 5 (11.9) | 0.443 |  |  |  |  |
|  | Lung disease | | | | 3 (1.2) | 3 (7.1) | 0.062 |  |  |  |  |
|  | Cardiovascular diseases | | | | 6 (2.5) | 1 (2.4) | >0.050 |  |  |  |  |
|  | Hepatobiliary disease | | | | 67 (27.8) | 11 (26.2) | 0.829 |  |  |  |  |
|  | Urinary system disease | | | | 21 (8.7) | 4 (9.5) | >0.050 |  |  |  |  |
|  | Nervous system disease | | | | 7 (2.9) | 1 (2.4) | >0.050 |  |  |  |  |
|  | Malignant solid tumor | | | | 52 (21.6) | 11 (26.2) | 0.507 |  |  |  |  |
|  | Hematological Disease | | | | 30 (12.4) | 5 (11.9) | 0.921 |  |  |  |  |
| Charlson comorbidity score ^b^(median, IQR) | | | | | 2 (0-2) | 2 (0-2) | 0.095 |  |  |  |  |
| Source of infections | | | | |  |  |  |  |  |  |  |
|  | | | Central venous catheterization | | 10 (4.1) | 0 (0) | 0.373 |  |  |  |  |
|  | | | Lung infection | | 41 (17.0) | 15 (35.7) | 0.005 | 0.083 | 2.012 | 0.912 | 4.437 |
|  | | | Abdominal infection | | 120 (49.8) | 21 (50.0) | 0.980 |  |  |  |  |
|  | | | Urinary infection | | 50 (20.7) | 8 (19.0) | 0.801 |  |  |  |  |
|  | | | Intracranial infection | | 3 (1.2) | 1 (2.4) | >0.050 |  |  |  |  |
|  | | | Skin infection | | 10 (4.1) | 1 (2.4) | 0.909 |  |  |  |  |
|  | | | Primary bloodstream infection | | 24 (10.0) | 2 (4.8) | 0.432 |  |  |  |  |
| Nosocomial- acquired infection | | | | | 150 (62.2) | 30 (71.4) | 0.253 |  |  |  |  |
| ICU stay after BSI ^c^ | | | | | 18 (7.5) | 5 (11.9) | 0.506 |  |  |  |  |
| Prior surgery ^a^ | | | | | 49 (20.3) | 7 (16.7) | 0.582 |  |  |  |  |
| Surgery after BSI ^c^ | | | | | 24 (10.0) | 0 (0) | 0.066 |  |  |  |  |
| Invasive procedure and/or devices prior to BSI ^a^ | | | | | 64 (26.6) | 19 (45.2) | 0.014 |  |  |  |  |
|  | | | Mechanical ventilation | | 8 (3.3) | 3 (7.1) | 0.453 |  |  |  |  |
|  | | | Central venous catheterization | | 32 (13.3) | 10 (23.8) | 0.076 |  |  |  |  |
|  | | | Urinary catheterization | | 33 (13.7) | 9 (21.4) | 0.193 |  |  |  |  |
|  | | | gastric catheterization | | 25 (10.4) | 6 (14.3) | 0.630 |  |  |  |  |
|  | | | Percutaneous catheterization | | 20 (8.3) | 1 (2.4) | 0.302 |  |  |  |  |
| Invasive procedure and/or devices after BSI ^c^ | | | | | 38 (15.8) | 9 (21.4) | 0.363 |  |  |  |  |
|  | | | Mechanical ventilation | | 17 (7.1) | 8 (19.0) | 0.026 |  |  |  |  |
|  | | | Central venous catheterization | | 68 (28.2) | 21 (50.0) | 0.005 |  |  |  |  |
|  | | | Urinary catheterization | | 63 (26.1) | 19 (45.2) | 0.012 | 0.097 | 1.879 | 0.891 | 3.963 |
|  | | | Gastric catheterization | | 47 (19.5) | 14 (33.3) | 0.044 |  |  |  |  |
|  | | | Percutaneous catheterization | | 31 (12.9) | 2 (4.8) | 0.212 |  |  |  |  |
| Corticosteroid use prior to BSI ^a^ | | | | | 16 (6.6) | 5 (11.9) | 0.377 |  |  |  |  |
| Corticosteroid use after BSI ^c^ | | | | | 18 (7.5) | 5 (11.9) | 0.506 |  |  |  |  |
| Prior Antibiotics use ^a^ | | | | | 107 (44.4) | 27 (64.3) | 0.017 | 0.100 | 1.872 | 0.886 | 3.954 |
|  | | Cepholosporins | | | 33 (13.7) | 5 (11.9) | 0.754 |  |  |  |  |
|  | | BLBLI combination antibiotics | | | 49 (20.3) | 13 (31.0) | 0.125 |  |  |  |  |
|  | | Tigecycline | | | 2 (0.8) | 1 (2.4) | 0.384 |  |  |  |  |
|  | | Carbapenems | | | 15 (6.2) | 5 (11.9) | 0.318 |  |  |  |  |
|  | | Aminoglycosides | | | 2 (0.8) | 0 (0) | >0.050 |  |  |  |  |
|  | | Quinolones | | | 30 (12.4) | 6 (14.3) | 0.724 |  |  |  |  |
| Laboratory examination ^a^ | | | | |  |  |  |  |  |  |  |
|  | | | White blood cell (median, IQR) | | 9.6 (5.7-14.8) | 10.1  (3.0-13.7) | 0.627 |  |  |  |  |
|  | | | Platelet (median, IQR) | | 121  (64-193) | 95 (42-188) | 0.359 |  |  |  |  |
|  | | | Total albumin (median, IQR) | | 32.3  (28.5-36.7) | 30.3 (23.5-35.9) | 0.045 | 0.045 | 0.941 | 0.887 | 0.999 |
| Severity of illness at time of BSI ^a^ | | | | |  |  |  |  |  |  |  |
|  | | APACHEII score (median, IQR) | | | 9 (6-12) | 13 (10-16) | <0.001 | 0.003 | 1.103 | 1.033 | 1.177 |
| Appropriate empirical treatment after BSI ^c^ | | | | | 216 (89.6) | 33 (78.6) | 0.042 | 0.058 | 2.526 | 0.968 | 6.593 |
|  | | 1. Cepholosporins | | | 18 (7.5) | 1 (2.4) | 0.378 |  |  |  |  |
|  | | 2. BLBLI combination antibiotics | | | 80 (33.2) | 19 (45.2) | 0.131 |  |  |  |  |
|  | | a. Monotherapy | | | 78 (32.4) | 17 (40.5) |  |  |  |  |  |
|  | | b. Combination therapy | | | 2 (0.8) | 2 (4.8) |  |  |  |  |  |
|  | | 3. Carbapenems | | | 110 (45.6) | 16 (38.1) | 0.364 |  |  |  |  |
|  | | a. Monotherapy | | | 102 (42.3) | 15 (35.7) |  |  |  |  |  |
|  | | b. Combination therapy | | | 8 (3.3) | 1 (2.4) |  |  |  |  |  |
|  | | 4. Quinolones | | | 25 (10.4) | 4 (9.5) | >0.050 |  |  |  |  |
|  | | 5. Aminoglycosides | | | 6 (2.5) | 1 (2.4) | >0.050 |  |  |  |  |
|  | | 6. Tigecycline | | | 2 (0.8) | 2 (4.8) | 0.106 |  |  |  |  |
|  | | 7.Other | | | 3 (1.2) | 1 (2.4) | >0..050 |  |  |  |  |
| Appropriate definitive treatments after BSI ^c^ | | | | | 230 (95.4) | 37 (88.1) | 0.124 |  |  |  |  |

Data are expressed as n (%) unless otherwise stated.

^a^ During the 30 days preceding BSI onset.

^b^ At time of BSI onset.

^c^ After BSI onset.

Abbreviations: ESBL, extended-spectrum beta-lactamase; *β-lactam-β-lactamase inhibitor* (BLBLI) combination antibiotics; APACHE, Acute Physiology and Chronic Health Evaluation; BSI, bloodstream infection; ICU, intensive care unit; IQR, interquartile range; SD, standard deviation.
